# Off-Label NOACs vs. Antiplatelets in AF-Related Stroke with GFR < 15 mL/Min/1.73 m^2^: A Multicenter Outcome Study

**DOI:** 10.3390/biomedicines13122927

**Published:** 2025-11-28

**Authors:** Jong-Hee Sohn, Minwoo Lee, Chulho Kim, Joo Hye Sung, Kyung-Ho Yu, Yerim Kim, Hee Jung Mo, Jae Jun Lee, Sang-Hwa Lee

**Affiliations:** 1 Department of Neurology, Chuncheon Sacred Heart Hospital, Hallym University College of Medicine, Chuncheon 24252, Republic of Korea; deepfoci@hallym.or.kr (J.-H.S.); gumdol52@naver.com (C.K.); centertruth@naver.com (J.H.S.); 2 Institute of New Frontier Research Team, Hallym University, Chuncheon 24252, Republic of Korea; 3 Department of Neurology, Hallym Sacred Heart Hospital, Hallym University College of Medicine, Anyang 14068, Republic of Korea; minwoo.lee.md@gmail.com (M.L.); ykh1030@hallym.or.kr (K.-H.Y.); 4 Department of Neurology, Kangdong Sacred Heart Hospital, Hallym University College of Medicine, Seoul 05355, Republic of Korea; brainyrk@gmail.com; 5 Department of Neurology, Inha University Hospital, Incheon 22332, Republic of Korea; hjungmo@gmail.com; 6 Department of Anesthesiology and Pain, Chuncheon Sacred Heart Hospital, Hallym University College of Medicine, Chuncheon 24253, Republic of Korea; iloveu59@hallym.or.kr

**Keywords:** antiplatelet, NOAC, AF-related stroke, ultra-low GFR, end-stage renal disease, dialysis, stroke recurrence, major bleeding

## Abstract

**Background:** This study aimed to evaluate the efficacy and safety of off-label use of non-vitamin K antagonist oral anticoagulants (NOACs) compared with antiplatelet therapy (APT) in patients with AF-related acute ischemic stroke (AIS) and a glomerular filtration rate (GFR) below 15 mL/min/1.73 m^2^. **Methods:** We used a multicenter prospective stroke registry to identify patients with AF-related AIS and GFR < 15 mL/min/1.73 m^2^ who were treated with either APT alone or NOAC alone at discharge. Primary outcomes were ischemic stroke recurrence, major bleeding, and all-cause mortality within one year. Adjusted hazard ratios (aHRs) and 95% confidence intervals (CIs) were calculated using Cox proportional hazards regression. **Results:** Among 311 eligible patients, 135 (43.4%) received APT and 176 (56.6%) received low-dose NOACs. Compared to APT, NOAC use was associated with a significantly lower risk of ischemic stroke recurrence (aHR 0.54, 95% CI 0.29–0.99) but higher risks of major bleeding (aHR 3.25, 95% CI 1.84–5.73) and all-cause mortality (aHR 2.65, 95% CI 1.60–4.38). The most common causes of death were non-vascular events such as sepsis and respiratory failure. **Conclusions:** In patients with AF-related stroke and ultra-low GFR, off-label use of NOACs may offer a benefit in stroke prevention but is associated with increased risks of bleeding and mortality. These findings suggest the need for individualized treatment strategies and careful monitoring when prescribing NOACs in this vulnerable population.

## 1. Introduction

Atrial fibrillation (AF) is a well-established risk factor for ischemic stroke, particularly due to its association with large vessel occlusion and more severe initial neurological deficits. The prevalence of AF increases as renal function declines, with studies reporting an AF prevalence of up to 23% in patients with a glomerular filtration rate (GFR) < 15 mL/min/1.73 m^2^ [1,2,3]. In patients with chronic kidney disease (CKD), especially those with end-stage renal disease (ESRD, GFR < 15 mL/min/1.73 m^2^), the coexistence of a prothrombotic and hemorrhagic milieu complicates stroke prevention strategies [4]. The use of antithrombotic agents in this population therefore requires careful consideration.

Non-vitamin K antagonist oral anticoagulants (NOACs) have emerged as effective alternatives to vitamin K antagonists (VKAs) for stroke prevention in patients with AF. However, their use in patients with advanced renal dysfunction remains controversial due to the potential for drug accumulation and heightened bleeding risk. As such, current clinical guidelines recommend against the use of NOACs in patients with GFR < 15 mL/min/1.73 m^2^ or those undergoing dialysis. Instead, VKAs remain the standard anticoagulant therapy in this group [5].

Despite guideline recommendations, VKA utilization in ESRD patients is limited due to the increased risk of bleeding, challenges with maintaining therapeutic INR levels, and complications such as calciphylaxis or vascular calcification [6,7,8]. Consequently, clinicians frequently opt for off-label use of NOACs, which offer advantages in dosing convenience and reduced monitoring requirements, or default to APT, despite its suboptimal efficacy in preventing cardioembolic stroke [9,10,11,12]. This trend is particularly pronounced in countries such as South Korea, where NOAC prescriptions have surpassed VKAs since 2018 [13]. However, direct comparative evidence between NOACs and APT in patients with AF-related stroke and ultra-low GFR remains scarce. Clarifying the clinical outcomes of these widely used but off-label strategies is crucial to optimizing treatment in this vulnerable group.

This study aimed to investigate the real-world prescription patterns of APT and off-label NOACs in patients with AF-related stroke and GFR < 15 mL/min/1.73 m^2^, and to evaluate their effects on stroke recurrence, major bleeding, and all-cause mortality.

## 2. Methods

### 2.1. Study Population

We consecutively enrolled patients with acute ischemic stroke in four university-affiliated registry databases between August 2013 and March 2023. We identified patient with non-valvular AF among cardioembolic strokes according to Trial of Org 10,172 an Acute Stroke Treatment (TOAST) classification with some modifications [14]. We included only those with an estimated GFR < 15 mL/min/1.73 m^2^ at admission. Exclusion criteria were: (1) absence of initial magnetic resonance imaging (MRI), (2) lack of one-year follow-up outcome data, (3) missing baseline laboratory results, and (4) prescription of warfarin or dual therapy with any type of oral anticoagulants and antiplatelets after discharge. Patients receiving combination antithrombotic therapy or with insufficient outcome data were excluded to avoid treatment classification bias and ensure accurate clinical outcome assessment.

### 2.2. Data Collection and Variable Definitions

Demographic, clinical, laboratory, and outcome data were extracted from web-based stroke registries at the participating hospitals. Non-valvular AF was confirmed during hospitalization via 24-h Holter monitoring and transthoracic echocardiography.

Renal function was assessed using the Chronic Kidney Disease Epidemiology Collaboration (CKD-EPI) formula to estimate GFR [15,16]. Although pivotal NOAC trials used the Cockcroft-Gault formula for renal dosing [5,17], most clinical laboratories in South Korea report estimated GFR based on CKD-EPI or the Modified Diet in Renal Disease (MDRD) formula [16]. The CKD-EPI equation has been used in a variety of populations, including kidney transplant patients in various populations [18,19]; therefore, we adopted it for this study. Dialysis status and modality were also confirmed via review of electronic medical records.

Antithrombotic therapy at discharge was categorized as either APT or NOACs. We defined APT at discharge as including aspirin, clopidogrel, cilostazol, and others. APT included agents such as aspirin, clopidogrel, and cilostazol, alone or in combination. NOACs included dabigatran, apixaban, rivaroxaban, and edoxaban. Patients were divided into two groups: those receiving APT alone and those receiving NOACs alone. The specific NOAC agent and dosage were confirmed by manual review of electronic prescriptions.

### 2.3. Outcome Measures

The primary outcome was ischemic stroke recurrence within one year. Secondary outcomes included major bleeding and all-cause mortality. Major bleeding was defined according to ISTH criteria as fatal bleeding, symptomatic bleeding in critical areas or organs (e.g., intracranial hemorrhage), or a ≥2 g/dL drop in hemoglobin or a transfusion of ≥2 units [20]. Patients were followed for one year after their index stroke. A multi-center institutional protocol was followed by stroke coordinators and nurses at each center to prospectively collect patient outcome data. This included the collection of modified Rankin Scale scores (mRS), incidence of death, cases of major bleeding, and other vascular events. These were monitored at three months and one year post-stroke through telephone interviews and comprehensive electronic medical record reviews.

### 2.4. Statistical Analysis

The patients were categorized into two groups based on their discharge prescriptions: those who were prescribed APT only and those who were prescribed NOAC only. To compare the demographic and clinical characteristics across the groups, Pearson’s chi-squared test was employed for categorical variables, while continuous variables were analyzed using either Student’s *t*-test or the Mann–Whitney U test.

To assess the influence of the APT and NOAC groups on primary and secondary outcomes, a Cox proportional hazard regression analysis was conducted, with the APT group serving as the reference point. This analysis was adjusted for clinically plausible covariates or those with univariate *p*-values less than 0.10. Both crude and adjusted odds ratios (ORs) and their 95% confidence intervals (CIs) were calculated. To estimate the event rates across antithrombotic groups, the Kaplan–Meier method was employed. Subsequently, the log-rank test was employed to ascertain the statistical significance of these rates between the groups.

A sensitivity analysis was conducted through propensity score matching (PSM). In this analysis, the propensity score for each patient, defined as the probability of receiving APT only or NOAC only based on initial demographics, vascular risk factors and laboratory tests was calculated using logistic regression. These cohorts were then matched in a 1:1 ratio using the nearest-neighbor method. Subsequent analysis using the two PSM cohorts involved cox proportional hazard regression to assess the effects of APT and NOAC on the outcomes. Covariate balance after PSM was evaluated using standardized mean differences and displayed as a Love plot (Supplementary Figure S1). To minimize overfitting given the limited number of events, we selected clinically relevant covariates a priori. A secondary Cox regression using a reduced model was performed.

To further evaluate the comparative effectiveness and safety of different antithrombotic strategies, we performed a subgroup analysis stratifying patients into three groups according to their discharge antithrombotic regimen: single antiplatelet therapy (SAPT), dual antiplatelet therapy (DAPT), and NOAC therapy. We used Kaplan–Meier survival curves and log-rank tests to compare cumulative event rates across the three groups. Additionally, multivariable Cox proportional hazards models were constructed, using SAPT as the reference group using for the total cohort.

All statistical analyses were conducted using IBM SPSS version 21.0 (IBM Corporation, Armonk, NY, USA) and MoonBook and MatchIt software (R version 4.0.3; R Core Team 2020, R Foundation for Statistical Computing, Vienna, Austria).

### 2.5. Ethical Statements

The collection of clinical information from the registry for the purpose of monitoring and improving the quality and outcomes of stroke care was performed with informed consent and was approved by the local institutional review board (IRB). (Approved by the Chuncheon Sacred Heart Hospital IRB, approval number 2013-03). The use of the registry database and the additional medical records for this study was approved by the IRB of the four centers without the need for informed consent from patients, due to its retrospective design and use of anonymized data. Thus the risk to patients was minimal. (Chuncheon Sacred Heart Hospital IRB number: 2024-09-001, approval date 24 September 2024; Hallym University Sacred Heart Hospital IRB number: 2024-09-008, approval date 24 September 2024; Dongtan Sacred Heart Hospital IRB number: 2025-02-006, approval date 26 February 2024 and Kangdong Sacred Heart Hospital IRB number: 2024-12-007, approval date 1 May 2025).

## 3. Results

**Figure 1:**
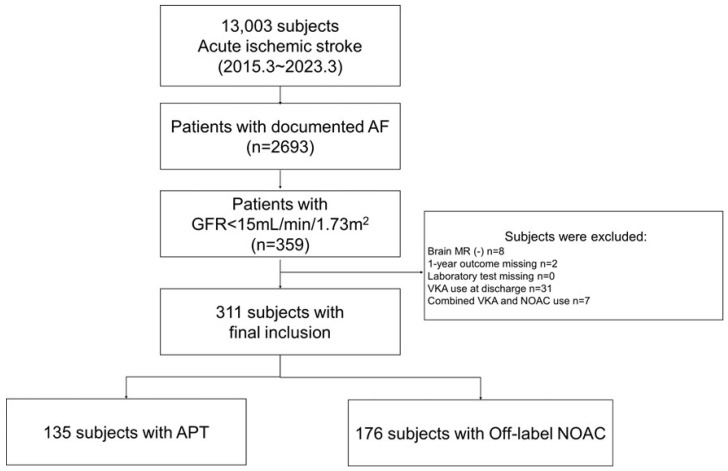
Flow chart of the study. Abbreviations: AF, atrial fibrillation; GFR, glomerular filtration rate; MR, magnetic resonance; VKA, vitamin K antagonist; NOAC, non-vitamin K antagonist; APT, antiplatelet therapy.

During the study period, 13,003 patients experienced acute ischemic stroke, of whom 2693 (20.7%) had a documented history of AF, diagnosed either before or after their index stroke. Of these, 311 patients (11.5%) met the inclusion criteria of having GFR < 15 mL/min/1.73 m^2^ at admission and being discharged on either APT alone or NOAC alone (Figure 1). Patients prescribed VKAs (*n* = 21) or combination therapy (VKA plus APT, *n* = 7) were excluded from the final analysis. The number of warfarin users was small and imbalanced. This study sought to evaluate antithrombotic strategies used in the real world when vitamin K antagonists (VKAs) are avoided in patients with severe renal dysfunction. Therefore, patients treated with VKAs were excluded. Consequently, this study reflects treatment patterns in settings where warfarin is not chosen due to concerns about bleeding, instability of the international normalized ratio (INR), or clinical preference. All patients were confirmed to have non-valvular AF based on echocardiographic evaluation. Of the final cohort, 135 (43.4%) received APT and 176 (56.6%) received low-dose NOACs. The distribution of prescribed NOACs was as follows: apixaban 2.5 mg (*n* = 73), edoxaban 30 mg (*n* = 69), rivaroxaban 15/10 mg (*n* = 25), and dabigatran 100 mg (*n* = 9) [21]. The selection of the NOAC dose and the decision to use it were based on the treating physicians’ clinical judgment rather than a uniform protocol due to ethical concerns in our cohort. All NOAC prescriptions in this study were reduced-dose regimens, reflecting cautious use in the setting of ultra-low GFR and high bleeding vulnerability.

Over one year, the overall rates of ischemic stroke recurrence and all-cause mortality were 14.7% and 31.2%, respectively. Vascular causes, including hemorrhagic events, accounted for approximately 20% of deaths. The leading causes of death were septic shock and respiratory failure. Baseline clinical characteristics were generally balanced between the two groups (Table 1). However, the NOAC group exhibited significantly higher rates of major bleeding (29.6% vs. 13.3%, *p* = 0.001) and all-cause mortality (42.0% vs. 17.0%, *p* < 0.001). Although the difference in stroke recurrence was not statistically significant by log-rank test (*p* = 0.10), a trend toward higher recurrence was observed in the APT group.

**Figure 2:**
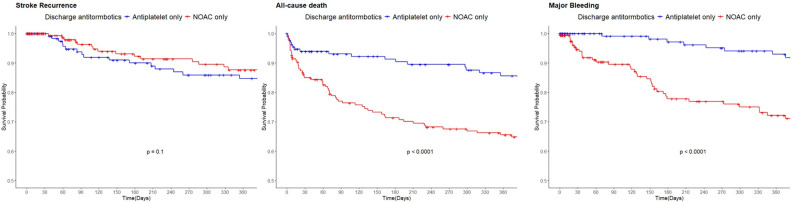
Kaplan–Meier Curve for Stroke Recurrence, Major Bleeding and All-cause Death According to the discharge antithrombotic status.

Multivariable Cox regression showed that NOAC use was independently associated with a lower risk of ischemic stroke recurrence (aHR 0.54, 95% CI 0.29–0.99), but higher risks of major bleeding (aHR 3.25, 95% CI 1.84–5.73) and all-cause mortality (aHR 2.65, 95% CI 1.60–4.38) (Table 2 and Supplementary Table S1). Kaplan–Meier analysis demonstrated significantly higher cumulative incidence of major bleeding and death in the NOAC group (Figure 2). In a sensitivity analysis using PSM, off label NOAC use was associated with higher bleeding and mortality, but the reduction in stroke recurrence (Table 2 and Supplementary Table S2).

In the three-group comparison (SAPT, DAPT, and NOAC), event-free survival differed significantly across antithrombotic strategies. For recurrent ischemic stroke, Kaplan–Meier analysis showed the greatest stroke-free survival in the NOAC group, followed by SAPT, with the lowest survival observed in the DAPT group (overall log-rank *p* = 0.004). In contrast, bleeding-free survival did not differ significantly (*p* = 0.18). For all-cause mortality, survival curves diverged significantly (*p* = 0.007), with DAPT demonstrating the highest survival, followed by NOAC and SAPT (Supplementary Figure S2). In addition, multivariable Cox models demonstrated concordant findings. Using SAPT as the reference group, NOAC therapy was associated with a lower risk of recurrent ischemic stroke, while both DAPT and NOAC showed numerically higher risks of major bleeding. Mortality risk was lowest in the DAPT group and higher in the NOAC group relative to SAPT, although this may reflect differences in baseline severity and treatment selection (Supplementary Table S3).

In sensitivity analyses using a reduced Cox model with fewer covariates, the direction and significance of the associations remained unchanged (Supplementary Table S4), indicating that overfitting was unlikely to affect the primary results.

## 4. Discussion

This study evaluated the comparative outcomes of APT and off-label NOACs in patients with AF-related acute ischemic stroke and GFR < 15 mL/min/1.73 m^2^. The key findings of this study are as follows: (1) over half of patients with AF-related stroke and GFR < 15 mL/min/1.73 m^2^ were prescribed off-label NOACs despite current guideline restrictions; (2) NOAC use was associated with reduced stroke recurrence; and (3) it was also associated with significantly increased risks of major bleeding and all-cause mortality compared to APT. Our findings provide important insights into the efficacy and safety of these therapeutic strategies in a population for which guideline-directed management is limited by the paucity of high-quality evidence.

The use of NOACs was associated with a significantly lower risk of ischemic stroke recurrence compared to APT in both total and PSM cohorts. This finding aligns with prior evidence supporting the superior efficacy of anticoagulants over antiplatelet agents in cardioembolic stroke prevention, even in patients with CKD [22,23]. Notably, all NOACs prescribed in our cohort were administered at low doses, likely reflecting clinicians’ cautious approach given the risks of drug accumulation in ESRD. This suggests that even low-dose NOACs may provide meaningful stroke risk reduction. A recent meta-analysis found that VKA did not improve recurrent ischemic stroke but increased the risk of hemorrhagic stroke in patients with AF and ESRD [7]. Based on our study, it is possible to consider the use of low-dose NOACs in patients with ESRD and AF who are at high risk of ischemic stroke recurrence compared to VKA. Although unmeasured confounding cannot be fully excluded due to the retrospective design, our PSM results suggest that preferential NOAC prescription to patients with higher embolic risk was unlikely. Specifically, there were no significant between-group differences after matching in key embolic risk indicators, including CHADS-VASc score, history of coronary heart disease, and dialysis status. These findings imply that clinicians did not selectively prescribe NOACs only to patients perceived to be at higher cardioembolic risk. Nevertheless, residual bias may remain, and our results should be interpreted as associative rather than causal. It should be noted that patients receiving VKAs were excluded from this study because of their small number and heterogeneous clinical profiles, which precluded meaningful comparison. Therefore, our findings do not suggest the superiority of NOACs over VKAs. Rather, they provide observational insights into the clinical outcomes of NOACs compared with antiplatelet therapy in real-world ESRD practice, where VKA use remains limited.

However, the benefit in stroke recurrence came at the cost of increased major bleeding (29.6% vs. 13.3%, *p* = 0.001). Among all major bleeding events, gastrointestinal bleeding was most common (44.1%), followed by intracranial (13.2%) and genitourinary (11.8%) sites. The distribution of bleeding sites showed a trend toward a difference between groups (*p* = 0.06, Supplementary Table S5)—gastrointestinal bleeding was more frequent in the NOAC group (55.3% vs. 30.0%), whereas transfusion-requiring events occurred more often in the APT group (40.0% vs. 10.5%). Although not statistically significant, these trends suggest that the two regimens may predispose to distinct bleeding profiles rather than differing solely in overall risk. Mechanistically, several overlapping factors may contribute to bleeding vulnerability in this population. Impaired renal clearance in ESRD can lead to NOAC accumulation even at reduced doses [24]; while uremic platelet dysfunction and altered fibrinolysis impair hemostasis and prolong bleeding time [25,26]. In addition, dialysis-induced platelet activation and endothelial stress may further destabilize vascular integrity [6,27]. Notably, most patients in this cohort (79.4%) were receiving dialysis, which is likely amplified these risks. Although the site distribution differences did not reach statistical significance, the observed pattern highlights the need for careful clinical surveillance for gastrointestinal bleeding, particularly in dialysis-dependent patients receiving NOACs. These overlapping risks may explain why NOACs led to a more than threefold increase in bleeding risk compared to APT (aHR 3.25; 95% CI, 1.84–5.73). Although prior meta-analyses have shown comparable bleeding risk between NOACs and VKAs [28], our findings support vigilant use and dosing adjustments when considering NOACs in patients with GFR < 15 mL/min/1.73 m^2^.

Although NOAC therapy significantly reduced the risk of recurrent ischemic stroke compared with antiplatelet regimens, it was paradoxically associated with higher all-cause mortality. This apparent contradiction can be explained by several clinical and mechanistic factors rather than by a direct harmful effect of NOACs themselves. First, most deaths in the NOAC group were nonvascular—primarily due to sepsis, respiratory failure, or general frailty—rather than fatal bleeding or recurrent stroke (approximately 21% pf causes of death, Supplementary Table S6). This indicates that excess mortality likely reflects differences in baseline vulnerability rather than treatment toxicity. Second, additional laboratory comparisons revealed that C-reactive protein (CRP) levels did not differ between groups, suggesting a similar baseline inflammatory burden, whereas serum albumin levels were lower in the NOAC group, implying poorer nutritional status and greater frailty. These findings support the interpretation that patients who were more physiologically compromised or nutritionally depleted were more likely to be prescribed NOACs, possibly because clinicians perceived them as having a higher embolic risk. Third, ESRD itself can amplify both bleeding and infection susceptibility through uremic platelet dysfunction, impaired drug clearance, and chronic inflammation, thereby increasing mortality even under appropriately dosed anticoagulation [25,29,30]. Furthermore, the short life expectancy and competing nonvascular risks in ESRD—most often driven by infection or cardiovascular collapse—may obscure the potential long-term thromboembolic benefit of NOACs. Therefore, the higher mortality observed in the NOAC group likely represents a composite effect of frailty-related vulnerability, patient selection bias, and dialysis-related nonvascular complications, rather than a direct adverse effect of the drug. Similar controversy surrounds VKA use and mortality in dialysis patients, with conflicting results across multiple observational studies [31,32,33]. The consistency of these findings, even in the reduced covariate Cox model designed to minimize overfitting, reinforces the robustness of our interpretation (Supplementary Table S6). Altogether, our results emphasize that the observed association between NOAC use and higher mortality should be interpreted with caution and highlight the need for larger, well-adjusted prospective studies to better inform clinical decision-making in this frail population. Our findings warrant validation through larger cohort studies or randomized controlled trials to better inform clinical decision-making.

In recent years, the use of NOACs among patients with end-stage renal disease (ESRD) has rapidly increased in South Korea, despite guideline recommendations that discourage their use in patients with GFR < 15 mL/min/1.73 m^2^. This real-world trend prompted the present study, which aimed to determine whether such off-label use of NOACs is justified in clinical practice. Our data confirmed that off-label NOAC prescribing is common among ESRD patients with AF-related stroke, reflecting a pragmatic response to the limitations of VKAs and the modest efficacy of APT. In the absence of high-level evidence, clinical decisions for ESRD patients with AF-related stroke increasingly rely on physician experience and individualized risk assessment rather than strict adherence to guidelines. Several factors contribute to this phenomenon. VKAs have limitations, including a narrow therapeutic index, INR variability, and an increased risk of calciphylaxis. NOACs are appealing due to their fixed dosing and lack of routine monitoring requirements. Furthermore, given the high one-year mortality rate among ESRD patients, stroke prevention strategies must balance efficacy with limited life expectancy. In this context, clinicians may prioritize preventing early recurrent strokes, even if it increases the risk of bleeding [12,34]. However, our findings also demonstrate that using NOACs in this population carries risks. Although NOAC therapy was associated with a lower risk of recurrent ischemic stroke, it also showed a tendency toward higher major bleeding and mortality. These results highlight that the growing real-world adoption of NOACs in ESRD patients should be approached with caution. In the absence of robust randomized data, choosing antithrombotic therapy in this setting must rely on an individualized, risk-stratified assessment that balances thromboembolic protection and bleeding risk. The increasing off-label use of NOACs thus reflects a pragmatic but imperfect solution to a clinical gap where evidence is scarce. Our study provides real-world data that clarifies the potential benefits and limitations of this practice, underscoring the urgent need for prospective trials to guide evidence-based treatment decisions in this vulnerable population.

The pharmacokinetics of NOACs are profoundly influenced by renal function. Approximately 80% of dabigatran, 50% of edoxaban, 35% of rivaroxaban, and 27% of apixaban are excreted by the kidneys in their active forms. In ESRD, reduced clearance leads to prolonged plasma half-lives and higher steady-state drug concentrations, even at reduced doses. Dialysis only removes a small amount of dabigatran and has a minimal effect on factor Xa inhibitors, such as apixaban, edoxaban, and rivaroxaban [35]. These alterations in pharmacokinetics can result in excessive anticoagulant exposure and an increased risk of bleeding, whereas diluting the dose too much out of caution may reduce the antithrombotic efficacy. In our cohort, apixaban and edoxaban were the most frequently used NOACs, likely reflecting physicians’ preference for factor Xa inhibitors with lower renal elimination fractions and a more favorable pharmacological profile in CKD. Furthermore, several real-world studies in Asian populations have reported that apixaban and edoxaban are associated with a lower risk of major bleeding compared with other NOACs, suggesting a potentially safer therapeutic window in this ethnic group [36]. These pharmacological and ethnic considerations may have contributed to their predominant use in our ESRD cohort. Nevertheless, our findings still demonstrated a higher rate of major bleeding among NOAC users, indicating that pharmacological advantages alone do not overcome the hemostatic fragility inherent to ESRD.

A clear pattern emerged when patients were stratified into SAPT, DAPT, and NOAC groups. NOACs provided the greatest reduction in recurrent ischemic stroke compared to SAPT. However, DAPT did not offer additional stroke prevention benefits and was associated with an increased risk of early recurrence. However, both the DAPT and NOAC groups showed increased bleeding tendencies relative to the SAPT group, with the DAPT group showing the highest risk, albeit numerically. Interestingly, the DAPT group had the lowest mortality rate, while the SAPT group had the highest, though this likely reflects treatment selection in real-world practice. NOACs are often chosen for patients with more aggressive stroke phenotypes, while SAPT is preferentially selected for patients who are frailer and have limited life expectancy. These findings reinforce the concept that antithrombotic therapy for patients with ultra-low GFR must be individualized. Although NOAC therapy may offer superior protection against thromboembolism, the increased risk of bleeding and mortality highlights the importance of careful patient selection and close clinical monitoring. The consistent directionality of these results across KM curves and adjusted Cox models strengthens the robustness of these observations.

A major strength of this study is that it addresses a critical gap in real-world data regarding antithrombotic strategies for AF-related stroke in patients with ultra-low GFR. The superior efficacy of NOACs in preventing stroke recurrence must be weighed against their increased bleeding risk and associated mortality in patients with AF-related stroke and GFR < 15 mL/min/1.73 m^2^. Our results may uniquely contribute robust real-world evidence on the comparative outcomes of these therapies, offering a nuanced understanding of their benefits and risks. Nonetheless, while this study provides valuable insights, certain limitations must be acknowledged. First, although PSM reduced baseline imbalances, hidden confounding may persist. Importantly, however, CHADS-VASc score, coronary heart disease history, and dialysis status did not differ between groups after matching, suggesting that NOACs were not preferentially administered to patients with higher embolic risk profiles. Despite these efforts, we acknowledge the possibility of unmeasured clinical judgment factors influencing treatment selection. Second, the exclusion of patients treated with VKAs and the focus on South Korean hospitals may limit the generalizability of findings to other populations and healthcare systems. Therefore, we are cautious to generalize our results. Third, the lack of standardized NOAC dosing protocols in the study population may have contributed to the observed bleeding complications. Fourth, the one-year follow-up period may not capture long-term outcomes, particularly the cumulative risks of bleeding and recurrent strokes. However, since over half of patients with ESRD died within one year of stating dialysis, it is considered that one-year outcomes are reasonable for such patients with several comorbidities [34]. Fifth, this study did not include pharmacokinetic or pharmacodynamic monitoring, such as measurement of plasma trough concentrations or anti–factor Xa activity. Consequently, direct assessment of NOAC accumulation or systemic exposure in ESRD was not possible. Future prospective trials incorporating therapeutic drug monitoring could help define optimal dosing strategies in this population. Lastly, we did not analyze the results according to the ingredients of NOAC. A previous meta-analysis revealed that apixaban and rivaroxaban are relatively safe in dialysis patients. However, the study’s novel aspect is that the majority of patients received edoxaban and apixaban [6]. The main ingredient of these NOACs may also be a factor in increased bleeding in our study. Future prospective studies with larger sample sizes should stratify outcomes by specific NOAC agents and dosages, as the pharmacodynamic profiles differ significantly among agents, particularly in the context of renal impairment.

In conclusion, among patients with AF-related stroke and GFR < 15 mL/min/1.73 m^2^, off-label NOAC use was associated with a lower rate of ischemic stroke recurrence, but a higher risk of major bleeding and mortality compared with antiplatelet therapy. These findings should be interpreted as observational associations rather than causal effects. Due to the retrospective design and potential residual confounding factors, prospective randomized studies are necessary to validate these results. In the meantime, clinicians should use an individualized, patient-centered approach when considering NOAC therapy for this vulnerable population.

## Figures and Tables

**Table 1:** Baseline characteristics according to the discharge antithrombotic therapy after AF-related stroke with GFR < 15 mL/min/1.73 m^2^.

	Total Cohort			PSM Cohort		
	APT Only (*n* = 135)	NOAC Only (*n* = 176)	*p*-Value	APT Only (*n* = 135)	NOAC Only (*n* = 135)	*p*-Value
Age, year (SD)	78.6 (10.1)	78.9 (9.8)	0.76	78.6 (10.1)	78.6 (10.1)	0.96
Male, *n* (%)	60 (44.4)	62 (35.2)	0.13	60 (44.4)	37 (27.4)	0.005
Body mass index, (SD)	23.1 (3.8)	22.7 (4.2)	0.39	23.1 (3.8)	22.3 (4.1)	0.14
Vascular Risk Factors						
Previous stroke, *n* (%)	43 (31.9)	54 (30.7)	0.92	43 (31.9)	43 (31.9)	1.00
Coronary heart disease, *n* (%)	34 (25.2)	43 (24.4)	0.98	34 (25.2)	34 (25.2)	1.00
Hypertension, *n* (%)	117 (86.7)	142 (80.7)	0.21	117 (86.7)	105 (77.8)	0.08
Diabetes, *n* (%)	59 (43.7)	96 (54.5)	0.08	59 (43.7)	86 (63.7)	0.002
Dyslipidemia, *n* (%)	36 (26.7)	36 (20.5)	0.25	36 (26.7)	22 (16.3)	0.054
Smoking, *n* (%)	16 (11.9)	22 (12.5)	1.00	16 (11.9)	16 (11.9)	1.00
Previous antiplatelets, *n* (%)	59 (43.7)	78 (44.3)	1.00	59 (43.7)	66 (48.9)	0.46
Previous anticoagulants, *n* (%)	32 (23.7)	50 (28.7)	0.39	32 (23.7)	37 (27.8)	0.53
Previous statin, *n* (%)	57 (42.5)	57 (32.4%)	0.09	57 (42.5)	48 (35.6)	0.29
Dialysis, *n* (%)	112 (83.0)	135 (76.7)	0.23	112 (83.0)	100 (74.1)	0.10
Laboratory findings						
Reperfusion therapy			0.67			0.49
None	106 (78.5)	141 (80.1)		106 (78.5%)	109 (80.7%)	
IVT	9 (6.7)	8 (4.5)		9 (6.7)	7 (5.2)	
EVT	15 (11.1)	17 (9.7)		15 (11.1)	10 (7.4)	
IVT plus EVT	5 (3.7)	10 (5.7)		5 (3.7)	9 (6.7)	
Hemoglobin, mg/mL (SD)	11.7 (2.5)	11.9 (2.6)	0.42	11.7 (2.5)	12.1 (2.7)	0.23
Creatinine, mg/mL (SD)	4.4 (1.5)	4.2 (1.3)	0.12	4.4 (1.5)	4.0 (0.9)	0.003
hemoglobin A1C, % (SD)	6.3 (1.3)	6.4 (1.5)	0.24	6.3 (1.3)	6.6 (1.7)	0.08
Fasting glucose, mg/mL (SD)	161.9 (75.9)	171.7 (91.0)	0.30	161.9 (75.9)	178.9 (96.8)	0.11
C-reactive protein, mg/L (SD)	23.9 (44.56)	23.3 (46.7)	0.86	25.9 (46.9)	26.5 (51.6)	0.70
Albumin, g/dL (SD)	4.07 (0.92)	4.08 (0.71)	0.01	4.12 (0.88)	4.05 (0.67)	0.01
systolic blood pressure, mmHg (SD)	146.2 (26.8)	141.7 (28.4)	0.16	146.2 (26.8)	139.0 (28.8)	0.04
low-density lipoprotein, mg/mL (SD)	84.5 (32.2)	86.1 (37.9)	0.70	84.5 (32.2)	85.7 (37.7)	0.79
CHA2DS2-VASc, score (SD)	5.2 (1.6)	5.3 (1.5)	0.37	5.2 (1.6)	5.5 (1.4)	0.053
HAS-BLED, score (SD)	4.3 (0.7)	4.4 (0.8)	0.84	4.3 (0.7)	4.4 (0.7)	0.36

Abbreviations: AF, atrial fibrillation; GFR, glomerular filtration rate; PSM, propensity score matching; APT, antiplatelet therapy; NOAC, non-vitamin K antagonist; SD, standard deviation; IVT, intravenous thrombolysis; EVT, endovascular treatment.

**Table 2:** Multivariable cox proportional-hazard regression analysis showing impact of off label use of NOAC on stroke outcome.

	Total Cohort		PSM Cohort	
	aHR	95% CI	aHR	95% CI
Stroke Recurrence	0.54	0.29–0.99	0.51	0.26–0.996
Major Bleeding	3.25	1.84–5.73	2.44	1.32–4.52
All-cause death	2.65	1.60–4.38	3.06	1.78–5.25

Abbreviation: NOAC, non-vitamin K antagonist; PSM, propensity score matching; aHR, adjusted hazard ratio; CI, confidence interval.

## Data Availability

All relevant data are available from the authors upon reasonable request.

## Supplementary Materials

The following supporting information can be downloaded at: https://www.mdpi.com/article/10.3390/biomedicines13122927/s1, Table S1: Multivariable cox proportional-hazard regression analysis showing impact of antithrombotic on stroke outcome using total cohort; Table S2: Multivariable cox proportional-hazard regression analysis showing impact of antithrombotic on stroke outcome using PSM cohort; Table S3: Multivariable cox proportional-hazard regression analysis showing impact of SAPT, DAPT and NOAC on stroke outcome using total cohort; Table S4: Sensitivity analysis using reduced covariate Cox proportional hazards models to address potential overfitting; Table S5: Distribution of major bleeding sites according to the ISTH definition in this study (*n* = 68); Table S6: Causes of death in this study (*n* = 97); Figure S1: Standardized mean differences of baseline covariates before and after propensity score matching. Vertical dashed line indicates an absolute standardized mean difference of 0.1, representing the threshold for acceptable covariate balance; Figure S2: Kaplan Meier Curve for Stroke Recurrence, Major Bleeding and All-cause Death According to the discharge SAPT, DAPT and NOAC.
