# Supplementary material for: Off-Label NOACs vs. Antiplatelets in AF-Related Stroke with GFR < 15 mL/Min/1.73 m2: A Multicenter Outcome Study
Source: Biomedicines. 2025 Nov 28;13(12):2927. doi: 10.3390/biomedicines13122927 (PMC12731138; doi:10.3390/biomedicines13122927)

Table S1. Multivariable cox proportional-hazard regression analysis showing impact of antithrombotic on stroke outcome using total cohort.

	Stroke Recurrence		Major Bleeding		All-cause death	
	aHR	95% CI	aHR	95% CI	aHR	95% CI
APT only	reference		reference		reference	
NOAC only	0.54	0.29-0.99	3.25	1.84-5.73	2.65	1.60-4.38
Age	1.03	0.99-1.07	1.03	1.00-1.06	1.01	0.98-1.04
Male sex	1.28	0.63-2.59	0.94	0.54-1.63	1.37	0.84-2.24
Body mass index	0.94	0.86-1.02	1.00	0.93-1.07	0.97	0.92-1.02
Initial NIHSS	0.92	0.86-0.98	1.02	0.98-1.07	1.08	1.05-1.11
Hypertension	1.09	0.39-3.02	0.88	0.40-1.95	0.63	0.35-1.14
DM	1.57	0.83-2.98	1.68	0.96-2.93	1.13	0.72-1.78
Dyslipidemia	0.47	0.19-1.14	0.54	0.27-1.10	1.05	0.63-1.76
Hemoglobin	0.98	0.86-1.12	0.87	0.79-0.96	0.94	0.86-1.01
Creatinine	0.91	0.65-1.28	1.24	1.06-1.46	0.86	0.68-1.09
Systolic blood pressures	1.01	1.00-1.02	1.00	0.99-1.01	1.00	0.99-1.00
Dialysis	1.83	0.76-4.40	1.38	0.69-2.77	0.62	0.36-1.09
CHA2DS2-VASc	0.96	0.75-1.23	0.94	0.77-1.16	1.07	0.91-1.25
HAS-BLED	1.26	0.79-2.01	1.20	0.83-1.75	1.03	0.76-1.41

Table S2. Multivariable cox proportional-hazard regression analysis showing impact of antithrombotic on stroke outcome using PSM cohort.

	Stroke Recurrence		Major Bleeding		All-cause death	
	aHR	95% CI	aHR	95% CI	aHR	95% CI
APT only	reference		reference		reference	
NOAC only	0.51	0.26-0.99	2.44	1.32-4.52	3.06	1.78-5.25
Age	1.02	0.99-1.06	1.01	0.98-1.04	1.02	0.99-1.05
Male sex	1.11	0.54-2.27	1.1	0.59-2.10	1.39	0.83-2.33
Body mass index	0.94	0.86-1.03	1.01	0.93-1.09	0.96	0.91-1.02
Initial NIHSS	0.91	0.85-0.97	0.99	0.96-1.04	0.85	0.45-1.58
Hypertension	1.52	0.51-4.52	1.15	0.50-2.67	0.85	0.63-1.64
DM	1.35	0.70-2.61	1.42	0.78-2.58	0.85	0.45-1.58
Dyslipidemia	0.32	0.11-0.92	0.58	0.26-1.30	0.89	0.49-1.62
CHA2DS2-VASc	0.93	0.72-1.21	0.99	0.79-1.23	1.06	0.89-1.26
HAS-BLED	1.20	0.73-1.99	1.01	0.66-1.53	0.90	0.65-1.25

Table S3. Multivariable cox proportional-hazard regression analysis showing impact of SAPT, DAPT and NOAC on stroke outcome using total cohort.

	Stroke Recurrence			Major Bleeding			All-cause death		
	aHR	95% CI	p-value	aHR	95% CI	p-value	aHR	95% CI	p-value
SAPT									
DAPT	1.298	0.62-2.70	0.485	1.772	0.85-3.70	0.128	0.429	0.20-0.93	0.032
NOAC	0.338	0.16-0.70	0.003	1.86	1.01-3.43	0.047	0.661	0.43-1.03	0.067
Age	0.983	0.95-1.02	0.295	1.007	0.98-1.04	0.635	1.058	1.03-1.09	<0.001
Male sex	0.877	0.40-1.91	0.742	1.844	0.96-3.53	0.065	0.923	0.55-1.56	0.765
Body mass index	0.946	0.87-1.03	0.195	0.998	0.93-1.07	0.956	0.975	0.92-1.03	0.36
Initial NIHSS	0.923	0.87-0.98	0.006	1.004	0.97-1.04	0.85	1.052	1.02-1.08	<0.001
Hypertension	1.089	0.41-2.90	0.864	1.003	0.45-2.25	0.994	0.613	0.34-1.10	0.1
DM	1.426	0.70-2.89	0.326	1.449	0.82-2.56	0.201	1.643	1.02-2.66	0.043
Dyslipidemia	0.534	0.22-1.29	0.163	0.516	0.27-1.00	0.05	0.916	0.55-1.52	0.732
CHA2DS2-VASc	1.014	0.72-1.42	0.935	1.159	0.89-1.52	0.281	0.856	0.69-1.06	0.154
HAS-BLED	1.262	0.79-2.03	0.334	1.019	0.69-1.50	0.924	1.145	0.83-1.57	0.404

Table S4. Sensitivity analysis using reduced covariate Cox proportional hazards models to address potential overfitting.

	Stroke recurrence			Major bleeding			All-cause death		
	HR	95% CI	p-value	HR	95% CI	p-value	HR	95% CI	p-value
APT									
NOAC	0.27	0.14-0.53	<0.001	1.42	1.84-2.41	0.03	1.76	1.15-1.54	0.01
Age	0.95	0.91-0.98	0.004	0.99	0.96-1.02	0.355	1.06	1.03-1.09	<0.001
Male	1.12	0.49-2.58	0.789	2.22	1.14-4.30	0.019	0.88	0.52-1.49	0.639
Initial NIHSS	0.92	0.87-0.97	0.004	1.001	0.97-1.04	0.947	1.05	1.03-1.08	<0.001
Dialysis	0.17	0.07-0.40	<0.001	0.39	0.20-0.74	0.004	0.86	0.82-1.45	0.58
CHA2DS2-VASc	1.20	0.84-1.72	0.326	1.25	0.95-1.64	0.106	0.88	0.71-1.09	0.246
HAS-BLED	1.41	0.87-2.29	0.162	1.04	0.70-1.55	0.835	1.14	0.84-1.57	0.401
Hypertension	0.78	0.28-2.13	0.622	0.85	0.38-1.92	0.696	0.55	0.31-0.98	0.042
DM	1.21	0.58-2.55	0.611	0.24	0.70-2.21	0.462	1.59	0.98-2.58	0.062

Table S5. Distribution of major bleeding sites according to the ISTH definition in this study (n=68).

	Overall	APT (n=30)*	NOAC (n=38)*
GI bleeding	30 (44.1%)	9 (30.0%)	21 (55.3%)
Transfusion	16 (23.5%)	12 (40.0%)	4 (10.5%)
Intracranial bleeding	9 (13.2%)	5 (16.7%)	4 (10.5%)
GU bleeding	8 (11.8%)	3 (10.0%)	5 (13.2%)
Pulmonary bleeding	4 (5.9%)	1 (3.3%)	3 (7.9%)
Others	1 (1.5%)	0 (0.0%)	1 (2.6%)

*p-value=0.06

Table S6. Causes of death in this study (n=97).

	Overall	APT (n=49)*	NOAC (n=48)*
Respiratory failure	38 (39.2%)	18 (36.7%)	20 (41.7%)
Septic shock	27 (27.8%)	11 (22.4%)	16 (33.3%)
Cardiac failure	8 (8.2%)	6 (12.2%)	2 (4.2%)
Cerebral infarction	15 (15.5%)	8 (16.3%)	7 (14.6%)
Cerebral hemorrhage	5 (5.2%)	3 (6.1%)	2 (4.2%)
Others	4 (4.1%)	3 (6.1%)	1 (2.1%)

*p-value=0.51

Figure S1. Standardized mean differences of baseline covariates before and after propensity score matching. Vertical dashed line indicates an absolute standardized mean difference of 0.1, representing the threshold for acceptable covariate balance.

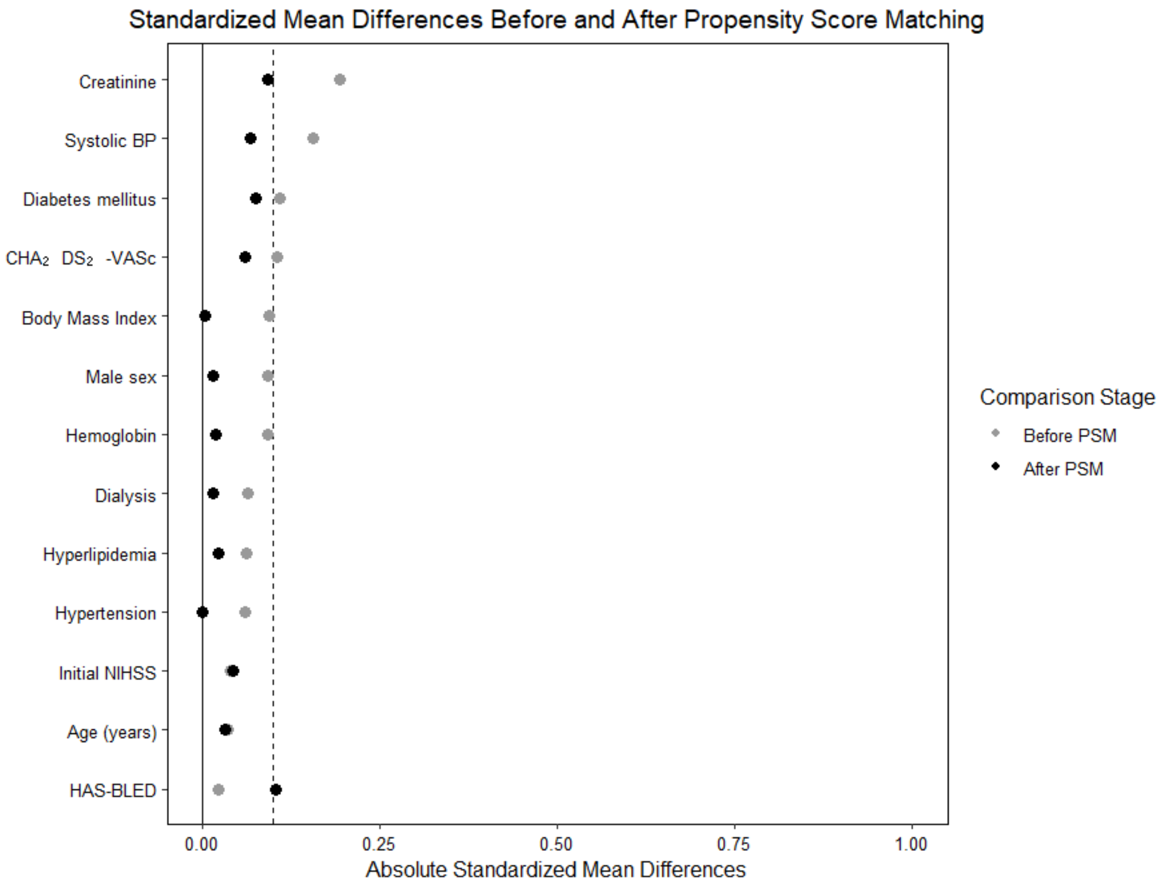

Figure S2. Kaplan Meier Curve for Stroke Recurrence, Major Bleeding and All-cause Death According to the discharge SAPT, DAPT and NOAC.

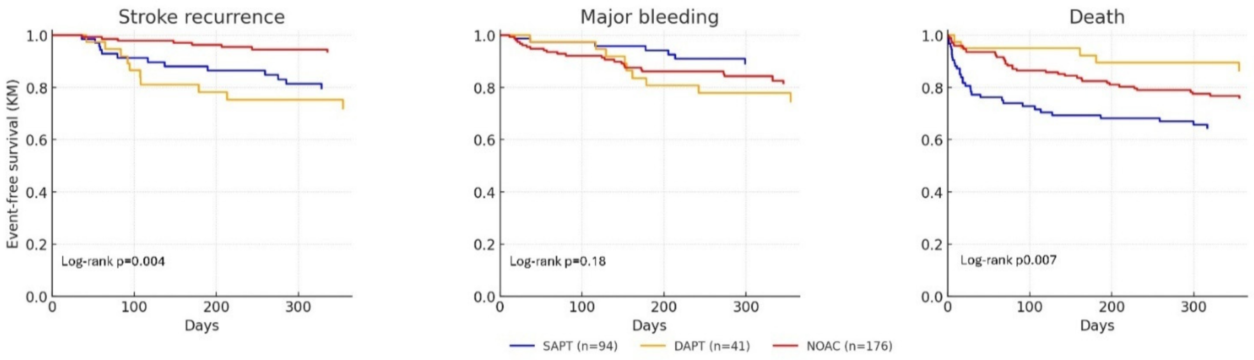

Supplement: Supplementary file 1 [file biomedicines-13-02927-s001.zip › biomedicines-3915086-supplementary.pdf]
